# Equitoxic Doses of 5-Azacytidine and 5-Aza-2′Deoxycytidine Induce Diverse Immediate and Overlapping Heritable Changes in the Transcriptome

**DOI:** 10.1371/journal.pone.0012994

**Published:** 2010-09-29

**Authors:** Xiangning Qiu, Christoffer Hother, Ulrik M. Ralfkiær, Alexandra Søgaard, Qianjin Lu, Christopher T. Workman, Gangning Liang, Peter A. Jones, Kirsten Grønbæk

**Affiliations:** 1 Department of Biochemistry and Molecular Biology, University of Southern California/Norris Comprehensive Cancer Center, Keck School of Medicine of the University of Southern California, Los Angeles, California, United States of America; 2 Department of Hematology, Rigshospitalet, Copenhagen, Denmark; 3 Center for Biological Sequence Analysis, Technical University of Denmark, Lyngby, Denmark; 4 Department of Dermatology, Second Xiangya Hospital, Central South University, Changsha, China; Wellcome Trust Centre for Stem Cell Research, United Kingdom

## Abstract

**Background:**

The hypomethylating agent 5-Azacytidine (5-Aza-CR) is the first drug to prolong overall survival in patients with myelodysplastic syndrome (MDS). Surprisingly, the deoxyribonucleoside analog 5-Aza-2′deoxycytidine (5-Aza-CdR) did not have a similar effect on survival in a large clinical trial. Both drugs are thought to exert their effects after incorporation into DNA by covalent binding of DNA methyltransferase (DNMT). While 5-Aza-CdR is incorporated into only DNA, 5-Aza-CR is also incorporated into RNA. Here, we have analyzed whether this difference in nucleic acid incorporation may influence the capacities of these drugs to regulate the expression of mRNA and microRNAs (miRNA), which may potentially affect the activities of the drugs in patients.

**Methodology/Principal Findings:**

A hematopoietic (HL-60; acute myeloid leukemia) and a solid (T24; transitional cell carcinoma) cancer cell line were treated with equitoxic doses of 5-Aza-CR and 5-Aza-CdR for 24 hrs, and the immediate (day 2) and lasting (day 8) effects on RNA expression examined. There was considerable overlap between the RNAs heritably upregulated by both drugs on day 8 but more RNAs were stably induced by the deoxy analog. Both drugs strongly induced expression of cancer testis antigens. On day 2 more RNAs were downregulated by 5-Aza-CR, particularly at higher doses. A remarkable downregulation of miRNAs and a significant upregulation of tRNA synthetases and other genes involved in amino acid metabolism was observed in T24 cells.

**Conclusions/Significance:**

Overall, this suggests that significant differences exist in the immediate action of the two drugs, however the dominant pattern of the lasting, and possible heritable changes, is overlapping.

## Introduction

The nucleoside analogs 5-Azacytidine (5-Aza-CR) and 5-Aza-2′-deoxycytidine (5-Aza-CdR) both have profound effects on the differentiated state of cultured cells [1]. It is generally assumed that this activity is related to the abilities of both drugs with an azanucleoside ring to be incorporated into replicating DNA, resulting in a powerful inhibition of DNA methyltransferases [2]. While 5-Aza-CdR is incorporated through a direct route into DNA, the ribose analog 5-Aza-CR must first be reduced at the diphosphate level by ribonucleotide reductase in order to be incorporated into DNA and hence inhibit DNA methylation. However a considerable portion of the drug can be incorporated into RNA where it could presumably influence RNA synthesis and metabolism. Nevertheless, the fact that both compounds are only active in the S phase of the cell cycle does imply a DNA linked mechanism of action [3].

Both drugs are approved by the US Food and Drug Administration (FDA) for the treatment of myelodysplastic syndrome (MDS), which is a malignant disease of the myeloid stem cells that primarily affects elderly patients. Hitherto, only high-dose myeloablative chemotherapy and allogeneic hematopoietic stem cell transplantation have shown a survival benefit in MDS patients, however this regimen is only feasible in the smaller fraction of younger MDS patients. Over the last decade clinical trials have established the efficacies of both drugs in causing disease remission in MDS and acute myeloid leukemia (AML) [4], [5], [6], [7], [8], [9], however two recent phase III clinical trials have shown that while 5-Aza-CR gave a distinct overall survival advantage to high risk MDS patients when compared to conventional care regimens [10], this was not seen in a trial conducted with the deoxy analog [11]. There are many potential reasons for this discrepancy, not the least of which is the different route of administration of the drugs, a different number of treatment cycles, and patient selection as demonstrated by differences in survival rates also in the control arms (15 months in the 5-Aza-CR study vs. 8,5 months in the 5-Aza-CdR study, respectively). Nonetheless, the result is surprising since both drugs do cause substantial decreases in DNA methylation, which is clearly associated with gene activation.

One remarkable difference between the two drugs is that 5-Aza-CR is incorporated into RNA in addition to its DNA related effects. The possibility therefore exists that this potential interference with RNA structure and function might be associated with different activities of the drugs in patients. We have therefore used high-throughput screening approaches to examine the effects of the drugs on mRNA and miRNA expression in an AML cell line (HL-60) and a solid tumor cell line (T24) at equitoxic doses of the drugs. We conducted our analyses at two time-points to detect immediate effects of the drugs on RNA expression and then repeated the analysis seven days after the drug had been removed from treated cells to compare and contrast heritable changes in gene expression induced by the two azanucleosides. The data show early changes in RNA synthesis, which might be due to RNA incorporation of the ribo analog yet clearly demonstrate the increased number of genes induced heritably by the deoxy analog 5-Aza-CdR.

## Materials and Methods

### Cell lines

The HL-60 cell line was originally derived from a female patient with AML-FAB M2 [12]. Its origin was confirmed by M-FISH (supplementary Fig. S1). The T24 cell line was derived from a female patient with urinary bladder transitional cell carcinoma. Its origin was confirmed by tandem repeat analysis (data not shown). HL-60 cells were cultured in RPMI 1640 medium with Glutamax-1 plus 10% FBS. T24 cells were cultured in McCoy's 5A medium supplemented with 10% FBS. To both cultures 100 units/ml penicillin and 100 µg/ml streptomycin was added.

### Drug treatment and determination of cell doubling times, cell cycling and apoptosis

To determine the optimal drug doses, HL-60 cells and T24 cells were treated with increasing concentrations of 5-Aza-CR or 5-Aza-CdR (Sigma-Aldrich, St. Louis, MO) for 24 h, the medium was changed, and cells were counted every 2 to 3 days with a Hemacytometer (Hausser Scientific, Horsham, PA) or a Z1 Coulter Particle Counter (Beckman Coulter Corporation, Hialeah, FL) and harvested on day 8. The equitoxic doses that led to approximately 50% prolongation in doubling time as compared to untreated cells were chosen for the microarray experiments. Apoptosis (Annexin V/FITC and Propidium Iodide) (BD Pharmingen, Franklin Lakes, NJ) and cell cycle analysis (EDU/7-AAD) assays (Invitrogen, Carlsbad, CA) were done to evaluate the effects of the drugs on apoptosis and cell cycling on day 2 and 8.

The cell cultures and drug doses used for subsequent microarray analysis were as follows: HL-60 cells were seeded at 5×10^5^ cells/25 cm^2^ flask (1×10^5^ cells/ml) 24 hrs prior to treatments and treated with 0.5 µM 5-Aza-CR or 0.1 µM 5-Aza-CdR (Sigma-Aldrich, St. Louis, MO). T24 Cells were plated at 3×10^5^ cells/100-mm dish and treated the next day with 30 µM 5-Aza-CR or 1 µM 5-Aza-CdR (Sigma-Aldrich, St. Louis, MO). The medium was changed after 24 hrs treatment, and cells were collected 1 day and 7 days after the drug had been removed. The cell number/flask or dish was counted every 2 to 3 days. Untreated cells were grown under similar conditions as a control. Two independent experiments were performed for each cell line.

### Nucleic acid extraction

Genomic DNA was collected with DNeasy Tissue Kit (Qiagen, Valencia, CA) according to the manufacturer's protocol. RNA was extracted using Trizol (Invitrogen, Carlsbad, CA) according to the manufacturer's instructions.

### Microarray analysis

A total number of 24 mRNA arrays and 13 miRNA arrays were analyzed. All mRNA arrays were run in biological and technical duplicates at each time point i.e. the following samples were analyzed: Six T24 samples (day 2: untreated/5-Aza-CR treated/5-Aza-CdR treated, and day 8: untreated/5-Aza-CR treated/5-Aza-CdR treated) and six HL-60 samples (day 2: untreated/5-Aza-CR treated/5-Aza-CdR treated, and day 8: untreated/5-Aza-CR treated/5-Aza-CdR treated). MiRNA arrays were analyzed in the same setup but in singlicate, (except for T24 on day 2 which was done in duplicate). Each miRNA-array contains 4 copies of each probe. All array data is MIAME compliant and the raw data has been deposited in ArrayExpress, (accession number E-MTAB-279/280).

#### Labeling and processing of mRNA data

Total RNA was labeled and hybridized to Illumina HG6 v3 beadarrays (Illumina, San Diego, CA) according to manufactures protocol. Two technical replicates were performed for each time point. Unnormalized summary data was exported from BeadStudio (Illumina, San Diego, CA) and imported into R/bioconductor using the BeadArray package, normalized using qspline [13] and batch corrected using a proprietary SVD correction. Data were annotated using http://www.compbio.group.cam.ac.uk/Resources/Annotation [14]. Probes designated as “Bad” were eliminated from further analysis.

#### Labeling and processing of microRNA

RNA was labeled according to manufactures protocol using miRCURY LNA™ microRNA Hy3/Hy5 Power labeling kit (Exiqon, Hørsholm, Denmark). RNA from HL-60/T24 was labeled with Hy3 and a mix of all samples was used as a common reference and labeled with Hy5. Five hundred ng of total RNA was hybridized on miRCURY LNA™ microRNA Array (Exiqon, Hørsholm, Denmark), v.11.0 using a Tecan HS4800 PRO (Tecan, Männedorf, Switzerland). Arrays were scanned on an Agilent scanner G2565CA (Agilent technologies, Santa Clara, CA) at 5 µm and 16 bit settings. Feature extraction was performed using GenePix Pro 6.1 (Molecular devices, Sunnyvale, CA) and Morphological background subtraction. Intensity values were imported into R 2.9.2 and analyzed using Limma [15]. The Lowess algorithm was used to normalize the expression values, and normalization between arrays was performed using Aquantile.

#### Detection of differential expression

Differential expression analysis was done using Limma [15]. First by fitting a linear model of the data to experimental design matrix and then by calculating Bayesian statistics. P-values were adjusted using the Benjamini-Hochberg correction.

#### Gene Set Enrichment Analysis (GSEA)

The technical replicates were collapsed into one using Limmas linear fit. Log 2 transformed expression values were annotated with HUGO symbols. These data were imported into javaGSEA desktop application v 2.0 [16], [17] where they were analyzed using 1000 permutations based on gene set, and signal2noise for ranking. Results were interpreted as positive if FDR corrected P-values were <0.05.

### Methylation-specific single nucleotide primer extension (Ms-SNuPE)

Two µg of each DNA sample was converted with EpiTect Bisulfite Kit (Qiagen, Valencia, CA) according to the manufacturer's protocol and each region of interest was amplified by PCR. The bisulfite specific PCR primer sequences for the *XAGE1D* promoter are as follows sense 5′-TTATATATAGAGAGGAGGGATTTT-3′ and antisense 5′-AACTCCCAATTAAATCTACCTA-3′. PCR conditions were as follow: 95°C for 5 min, followed by 95°C for 1 min, 55°C; 1 min, 72°C; 1 min, for a total of 40 cycles. The PCR amplicons were extracted with the Gel Extraction Kit (Qiagen, Valencia, CA), and Ms-SNuPE analysis was performed to examine the methylation level changes as previously described [18]. Primers used for Ms-SNuPE analysis were 5′-TTTTTTGGTGTTTATTTTAGTG-3′ and 5′- GTTAGTGTGGGGAA-3′. PCR conditions: One cycle of 95°C for 1 min, 50°C for 1 min, 72°C for 1 min.

### Methylation specific melting curve analysis (Ms-MCA)

For methylation screening of the candidate tumor suppressor gene (TSG) *BTG2* we used Ms-MCA as described [19]. Amplification was carried out on a LightCycler 480 instrument (Roche diagnostics, Basel, Switzerland) using the Fast Start SYBR green I Kit (Roche diagnostics, Basel, Switzerland), sense primer 5′- GGGTAAgGTTGTTTTGTGGATT-3′ and anti-sense primer 5′-TTTTTATTcGAGATTTTTTATTGAGTT. The PCR conditions were as follows: 5 min of initial enzyme activation at 95°C, 45 cycles of denaturation at 95°C for 10 s, annealing at 60°C for 20 s and elongation 72°C for 30 s. Subsequently, melting curves were obtained by measuring the drop in fluorescence when raising the temperature from 67 to 98°C with 5 observations per second. The melting peaks were calculated using the LightCycler 480 Software Release 1.5.0SP3.

### Quantitative real-time PCR

Reverse transcription (RT) was performed with M-MLV reverse transcriptase and random hexamers (Promega, Madison, WI) according to the manufacturer's instructions. Quantitative real-time PCR for XAGE1D was done using TaqMan probes (Biosearch Technologies, Novato, CA): sense primer 5′-TCCCCAGACGGGACCAG-3′ and antisense primer 5′- CTGGCTGTGTGGTTCTGTGTTT -3′, probe 5′- FAM-AGAGGGACGGCATGAGCGACACAC-BHQ-3′. The real-time RT-PCR conditions were as follows: 95°C for 9 min, followed by 45 cycles of 95°C for 15 s and 60°C for 1 min. Confirmatory RT-qPCR analysis of other CTAs and tumor suppressors was done on the Light Cycler. For primers and conditions see supplementary Table S1.

## Results

**Figure 1:**
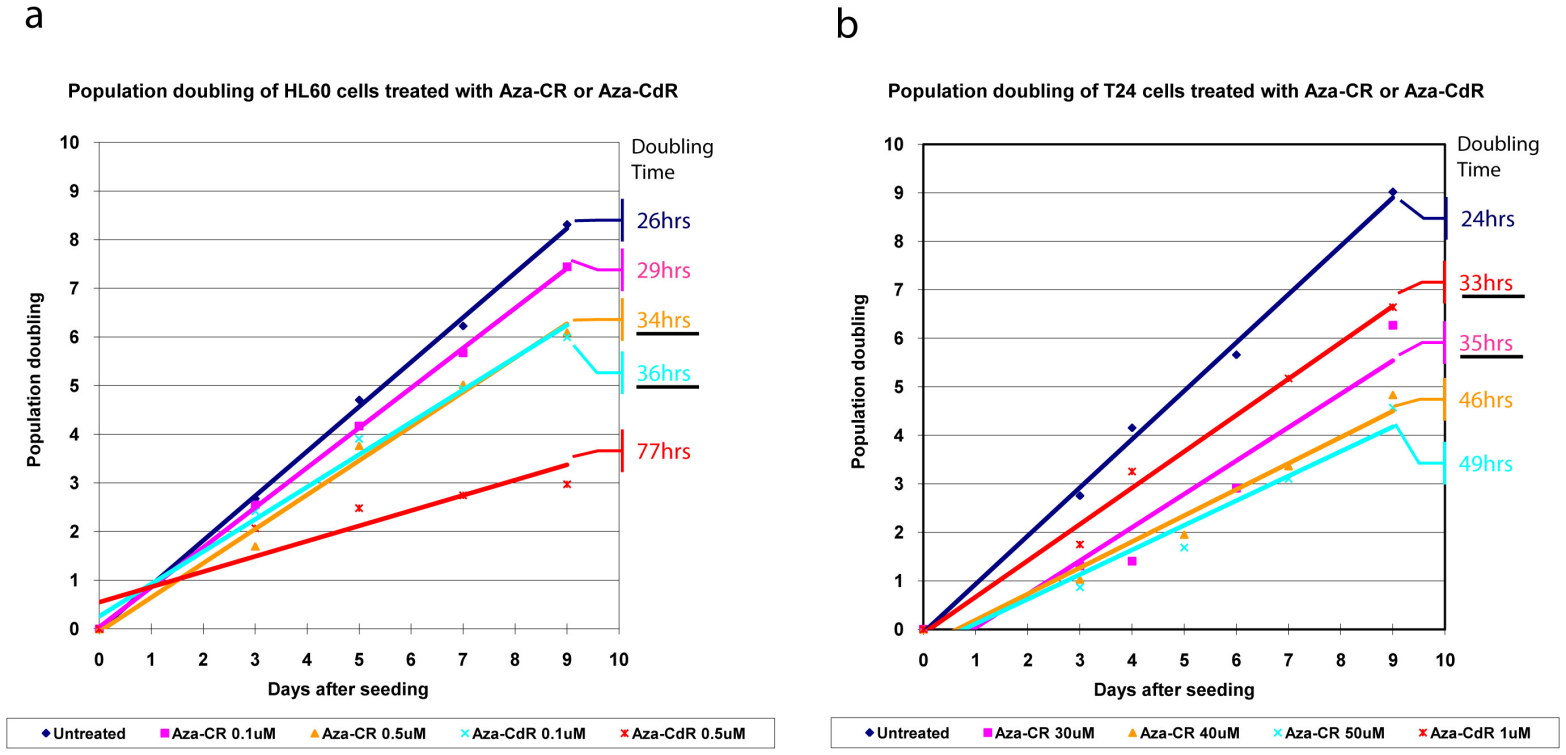
Estimation of population doubling times in the HL-60 and the T24 cell lines. Population doubling (PD) curves of the HL-60 (a) and (b) T24 cell line. Levels of cellular growth after treatment, with the indicated drug regimens plotted as population doublings against time. Initial drug treatment was started 24 h after seeding. The lines are trend lines.

The optimal doses of 5-Aza-CR and 5-Aza-CdR were determined by monitoring the effects of increasing doses of both compounds on the doubling times of the two cell lines (Fig. 1). “Equitoxic dose” was defined as the dose of both drugs leading to similar doubling time changes and doses were picked that increased the doubling time by approximately 50%. The two cell lines showed considerable differences in drug sensitivity, in favor of the myeloid cell line. The doses were 0.5 µM 5-Aza-CR and 0.1 µM 5-Aza-CdR for the HL-60 cells and 30 µM 5-Aza-CR and 1 µM 5-Aza-CdR for the T24 cells. At the chosen doses, no significant differences between the two drugs were observed with regards to the fraction of cells being in S-phase and apoptosis at day 2 and 8 respectively. Notably, the high dose of 5-Aza-CR used in the T24 cells did not cause increased apoptosis. In addition, <20% apoptosis was observed in both cell lines at the chosen doses at both time points (supplementary Fig. S2).

### Changes in mRNA expression

**Figure 2:**
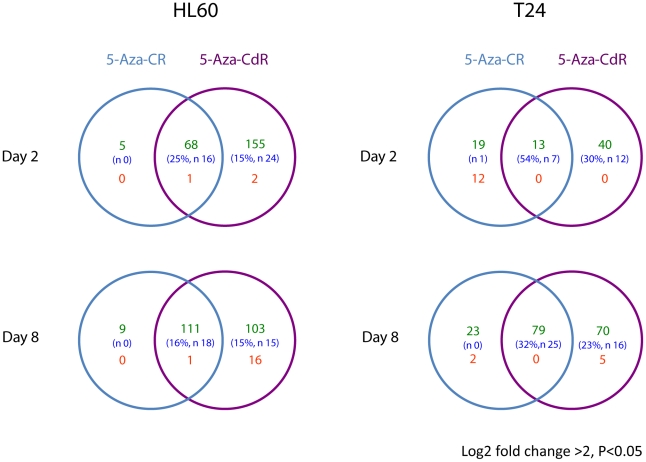
mRNA expression after treatment with 5-Aza-CR and 5-Aza-CdR. Venn diagrams showing differentially expressed transcripts in the HL-60 AML cell line and the T24 bladder cancer cell line harvested and analyzed 1 and 7 days after treatment with equitoxic doses of 5-Aza-CR and 5-Aza-CdR. The numbers in each area show genes differentially expressed in treated relative to untreated cells. Upregulated genes are shown in green, downregulated genes in red. Numbers of upregulated CTAs, and the percentage they comprise of the total number of upregulated genes, are indicated in blue.

Both drugs caused significant upregulation of a large number of genes in the HL-60 cells on day 2 (Fig. 2). Although there was a considerable overlap in the gene sets upregulated, 5-Aza-CdR was clearly more effective at gene induction in that it induced expression of 155 genes that did not overlap with genes upregulated by the ribo analog. Neither drug caused substantial downregulation of genes at the stringent cut off value (log2 fold change >2). Interestingly, the overlap between the gene sets increased on day 8, which is consistent with both drugs acting predominantly through a heritable epigenetic process involving DNA methylation. The fact that 5-Aza-CdR activated more genes and that only 9 genes were exclusively upregulated by 5-Aza-CR on day 8 again points to the same mechanism of action. The few genes that were downregulated at day 8 were probably influenced by a negative regulatory process due to the switching on of a subset of the 214 genes heritably activated by 5-Aza-CdR.

The small number of genes immediately downregulated on day 2 by 5-Aza-CR in the HL-60 cells suggested that little drug was being incorporated into RNA, where it might influence the stability and/or processing of mRNA. This situation was different in T24 cells where 12 mRNAs were immediately downregulated by the ribo- but not the deoxyribo analog (Fig. 2). These changes were not permanent and largely disappeared on day 8. More genes were upregulated at both time points exclusively by 5-Aza-CR in the T24 cells than in the HL-60 cells. Overall the data show considerable overlap of the gene sets heritably altered by both drugs in both cell types with 5-Aza-CdR being the most effective. On the other hand, the fact that a unique set of genes was altered by the ribo analog in T24 cells on day 8 suggests that interference with RNA metabolism during drug treatment can also induce heritable effects on gene expression.

### Common pathways upregulated by the two drugs

A more detailed GSEA of the mRNA expression data shows that both drugs deregulate a set of genes, which are also deregulated in *Dnmt1* KO mouse cells [20] (see supplementary Table S2). However, quite surprisingly, none of the TSGs known to be inactivated by promoter methylation in the myeloid cell line (*HIC-1, CDH1* and *ER*) [21] or in the T24 cell line (*CDNK2A* [18] and *RUNX3* [22]) was upregulated to significant levels by either of the drugs. So, although previous studies have shown that these types of changes are detectable by PCR based methods, the level of upregulation of these genes was not large enough to be detected by the arrays.

Interestingly, TSGs that have not been found methylated in AML showed significant upregulation by both drugs in HL-60 with the highest upregulation induced by 5-Aza-CdR (supplementary Fig. S3). These include critical cell cycle regulating tumor suppressors such as cyclin dependent kinase inhibitors CDKN1C (p57) and the cell cycle and differentiation associated BTG2. Since the candidate TSG *BTG2* has not previously been analyzed for promoter methylation in AML, we performed methylation scanning by Ms-MCA, but saw no methylation, indicating that different mechanisms may have caused upregulation of BTG2. It has been known for sometime that 5-Aza-CdR can turn on unmethylated genes in addition to methylated genes [23].

#### Cancer testis antigens

**Figure 3:**
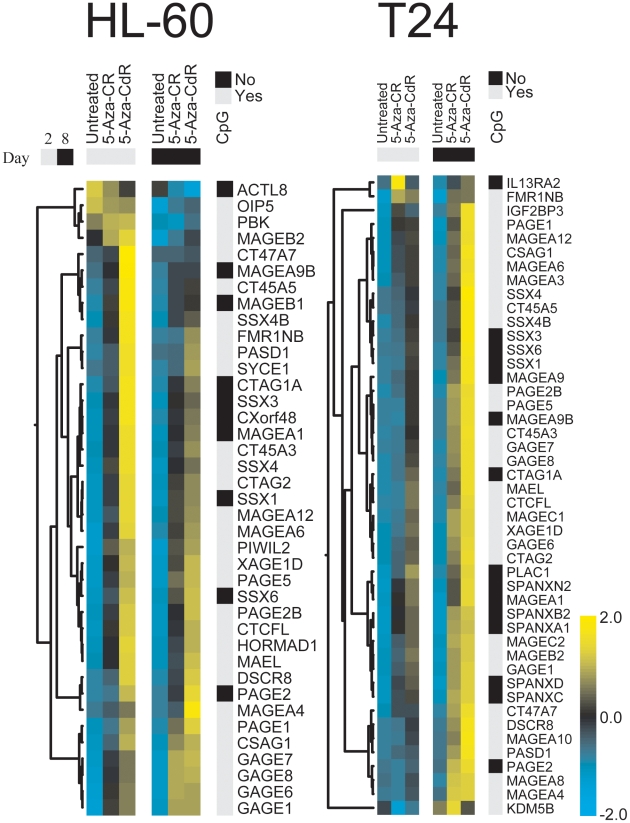
Expression of cancer testis antigens in azanucleoside treated and untreated HL-60 and T24 cell lines. Heatmaps showing CTAs that vary across treatment conditions (SD>0.25). The heatmaps were generated from the 83 CTAs present on the arrays based on genes selected from the CTpedia database http://www.cta.lncc.br/index.php. Genes that did not vary were removed, leaving 39 CTAs in HL-60 cells and 46 CTAs in T24 cells. The linear fits from the Limma analysis were used to generate heatmaps. Data were clustered based on Euclidian distance and each row were normalized using z-score. CpG islands were identified using www.cpgislands.com with standard settings except for length >200 bp.

A notable proportion of the genes commonly upregulated by both drugs in both cell lines belong to the group of cancer testis antigens (CTA; Fig. 2). All but one CTA upregulated by 5-Aza-CR were also upregulated by 5-Aza-CdR, which also upregulated additional CTAs. These antigens were upregulated in HL-60 cells by both drugs already on day 2, and the level of expression increased on day 8 for 5-Aza-CR but decreased for 5-Aza-CdR treated cells. Hardly any expression was induced in T24 cells on day 2 by 5-Aza-CR and for both drugs the level of expression was highest on day 8 (Fig. 3).

**Figure 4:**
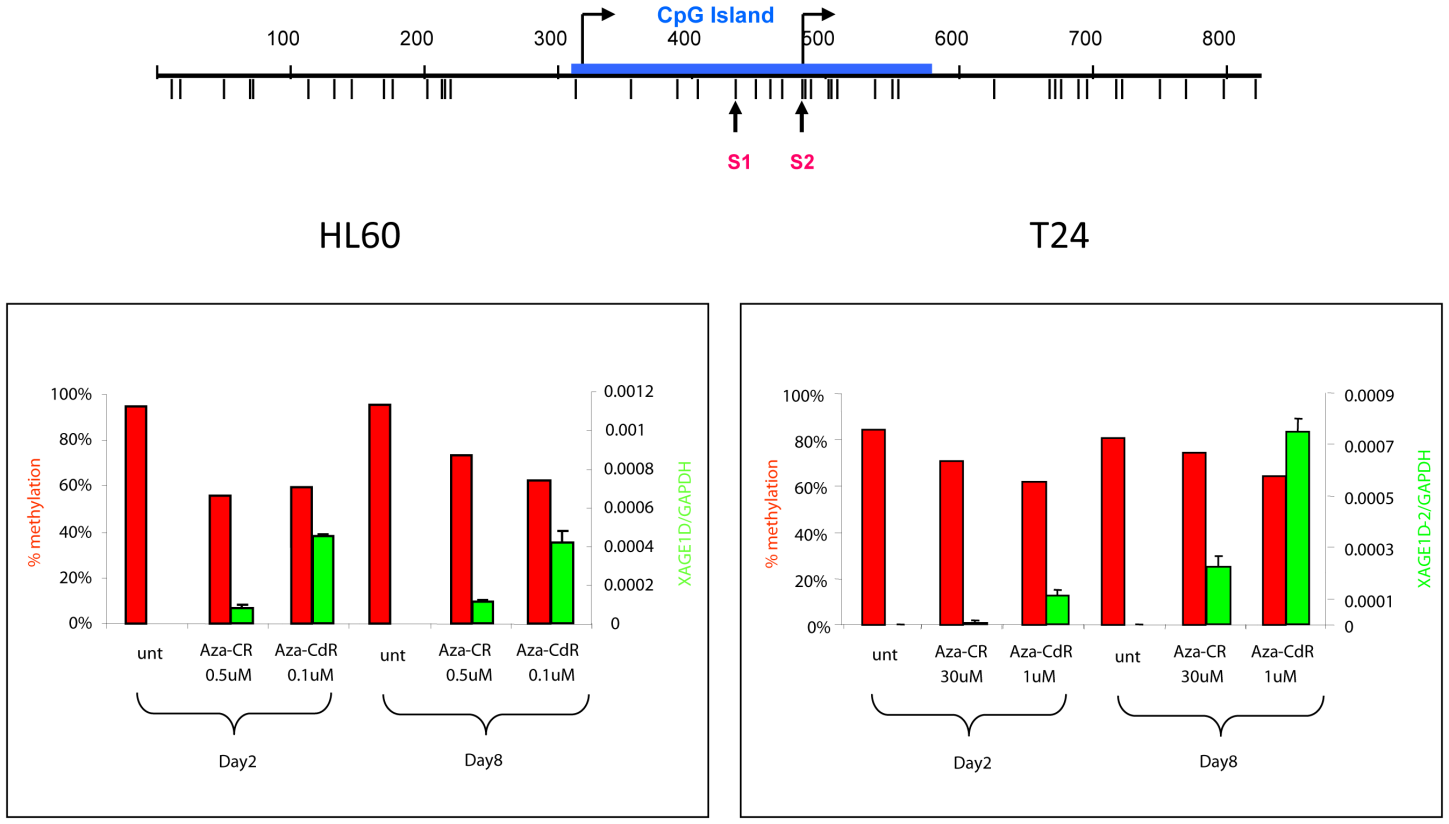
Induction of expression of XAGE1D by 5-Aza-CR and 5-Aza-CdR. Upper: Schematic drawing of the XAGE1D promoter CpG island. CpG sites analyzed by Ms-SNuPE are indicated by the arrows. Lower: Level of promoter methylation estimated by Ms-SNuPE relative to the level of mRNA expression estimated by RT-qPCR at equitoxic doses of 5-Aza-CR and 5-Aza-CdR on day 2 and day 8 in the HL-60 and the T24 cells.

A proportion of CTA genes have CpG islands in their promoters (Fig. 3), and they are likely to be upregulated by promoter demethylation (Fig. 4). A detailed analysis of the *XAGE1D* promoter showed that this promoter is 95% hypermethylated in the untreated HL-60 cell line. A 40% reduction of methylation was induced by both drugs, however 5-Aza-CdR caused approximately 5 times higher expression than 5-Aza-CR at both time points. No significant increase in expression from day 2 to day 8 was seen in this cell line. In T24 cells, 5-Aza-CdR also led to a higher expression level than did 5-Aza-CR. Hardly any expression was induced on day 2 by 5-Aza-CR, and a higher expression level was achieved on day 8 for both drugs (Fig. 4). Similar results were obtained by detailed RT-qPCR analyses of different CTAs (supplementary Fig. S4). Since not all CTAs have a CpG island in their promoters, we speculated whether other factors might have induced the high expression of CTAs. Interestingly, the CTCF paralogue CTCFL/BORIS, that can reactivate the transcription of other CTAs [24], was also upregulated by both drugs in both cell lines. This gene has CpG islands in 2 of its 3 promoters and 5-Aza-CdR has previously been shown to strongly upregulate transcription by mechanisms both dependent and independent of DNA methylation [25]. In HL-60 cells CTCFL/BORIS showed significant upregulation already on day 2, and its expression coincided with CTA upregulation. CTCFL/BORIS is believed to antagonize CTCF and in this cell line, CTCF was downregulated at each time point by both drugs. Only a minor fraction of the CTAs was upregulated by 5-Aza-CR on day 2 with no upregulation of CTCFL/BORIS in T24 cells. More upregulation of both CTCFL/BORIS and other CTAs was seen by 5-Aza-CdR. On day 8 both drugs showed high upregulation of a large proportion of CTAs including CTCFL/BORIS (Fig. 3).

#### Inflammation and immune modulating pathways

GSEA also showed that commonly regulated pathways involved in inflammation and immune mechanisms dominate (supplementary Table S2). These involve activation of genes implicated in cytokine signaling including members of the interleukin-, interferon- and TNF receptor families. The upregulation of these pathways was seen by both drugs on both days in HL-60 cells but only on day 8 in T24 cells. Taken together, the CTAs and immunomodulatory pathways are commonly upregulated by both drugs, particularly on day 8, indicating that the heritable changes in gene expression are similar for the two drugs.

### Diverse pathway regulation by the two drugs

Since not many differences were seen in the heritable changes in gene expression, we wondered if a diverse expression pattern is seen early after treatment as has been suggested by others [26]. The most striking difference between the two drugs was that 5-Aza-CdR upregulated a large number of genes that are not expressed after 5-Aza-CR treatment. Interestingly, GSEA shows that several gene sets related to MYC, MYB and E2F oncogene signaling are enriched by 5-Aza-CdR treatment on day 2 in both cell lines, indicating an immediate activation of growth promoting pathways by this drug (supplementary Table S2). However, since we have demonstrated that growth is inhibited in both cell lines at this point (Fig. 1 and supplementary Fig. S2) the upregulation may be transient or compensatory and therefore biologically irrelevant. Furthermore, particularly in T24 cells a few gene sets were upregulated more by 5-Aza-CR than by 5-Aza-CdR as described below.

#### Diverse pathways deregulated by the two drugs in the T24 cell line

**Figure 5:**
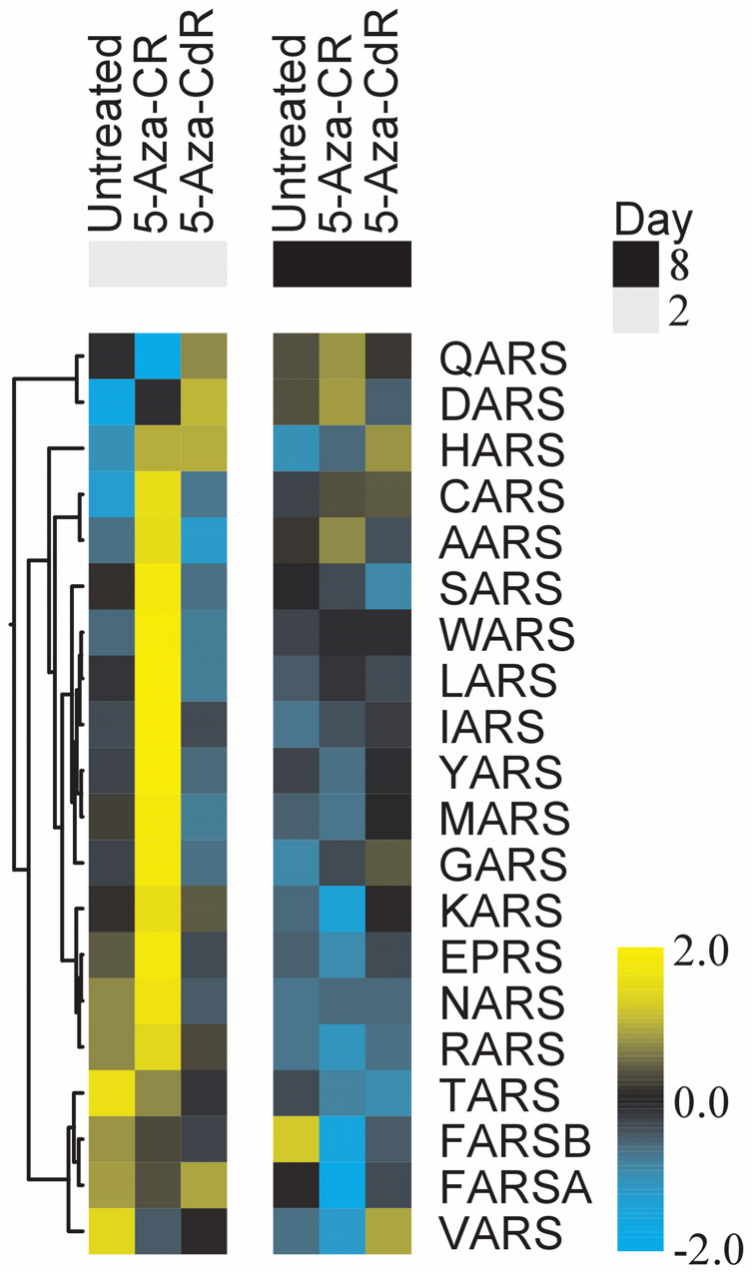
Hierarchical clustering showing differential expression of tRNA synthetase genes. Heatmap of the linear fits from the Limma analysis, showing tRNA synthetase genes primarily upregulated in 5-Aza-CR treated T24 cells on day 2.

One striking observation in T24 cells was that a large number of tRNA synthetase genes (13/20 = 65% of all tRNA synthetase genes) became upregulated to a significant level (p<0.05) on day 2 by 5-Aza-CR but not by 5-Aza-CdR (Fig. 5). This became even more pronounced when the cells were treated with higher doses of 5-Aza-CR (50 µM) (data not shown). This upregulation disappeared on day 8, where a general downregulation of the tRNA synthetase genes was seen by both drugs in both cell lines. Interestingly, GSEA showed that several pathways associated with amino acid synthesis and metabolism were deregulated on day 2 by 5-Aza-CR (supplementary Table S2).

#### Effects of the two azanucleosides on miRNA expression

**Figure 6:**
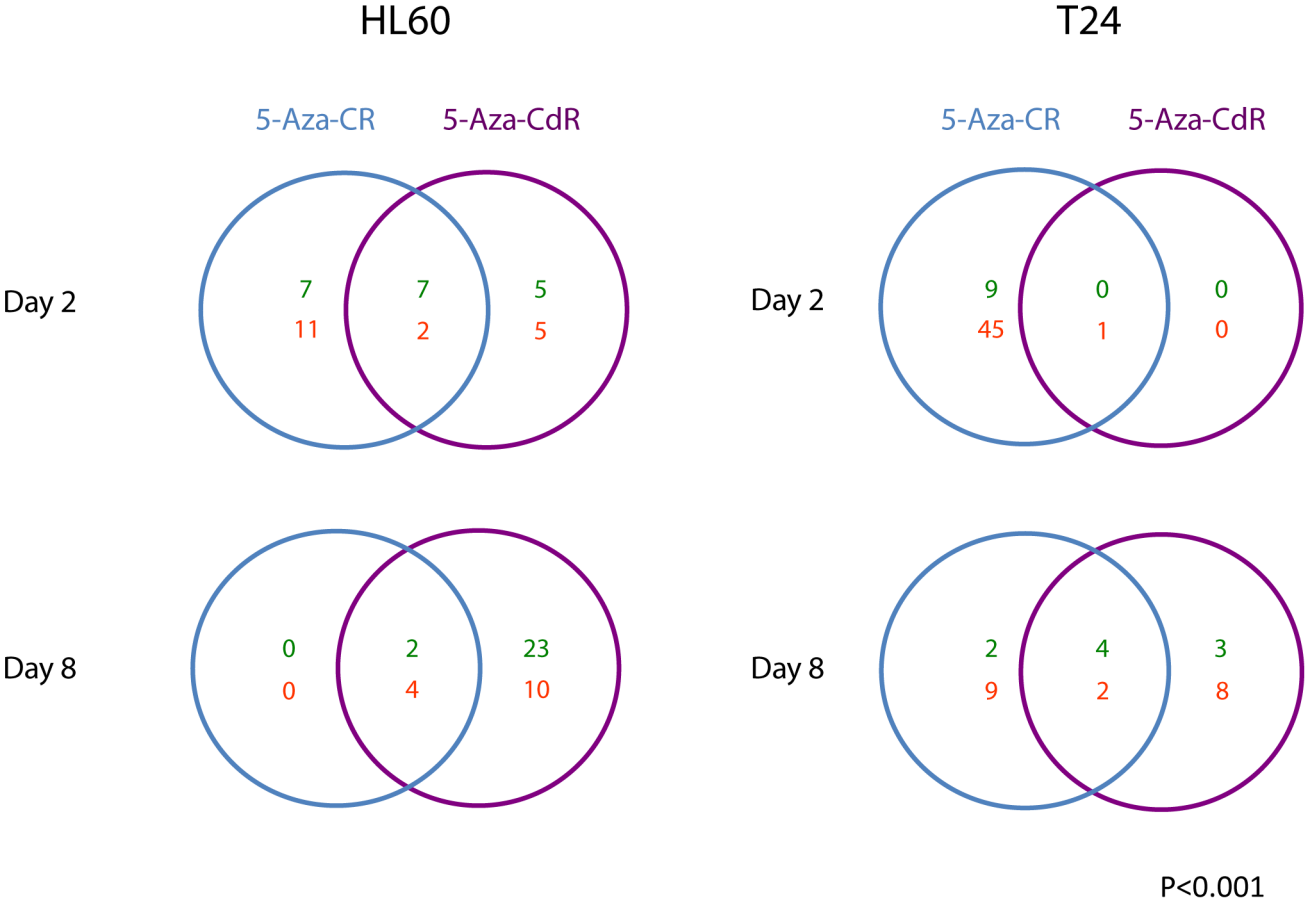
miRNA expression after treatment with 5-Aza-CR and 5-Aza-CdR. Venn diagrams showing differentially expressed miRNAs in the HL-60 AML cell line and the T24 bladder cancer cell line harvested and analyzed 1 and 7 days after treatment with equitoxic doses of 5-Aza-CR and 5-Aza-CdR. The numbers in each area show miRNAs differentially expressed in treated relative to untreated cells. Upregulated miRNA are shown in green, downregulated miRNAs in red.

Since earlier results have shown that miRNAs are subject to epigenetic control [27], [28], [29], it was essential to compare and contrast the effects of the 2 drugs on miRNA expression. In general, the altered expression patterns for miRNAs were different from what was observed for mRNAs (Fig. 6).

On day two, 5-Aza-CR caused the most dramatic changes with significant miRNA downregulation in both cell lines, which might be a result of incorporation of the ribo analog into newly synthesized miRNA molecules. In HL-60 cells 13 miRNAs were down- and 14 upregulated, whereas in the T24 cell line as many as 46 miRNAs were down- and 9 upregulated immediately after treatment. In contrast, the deoxyanalog (5-Aza-CdR) caused less downregulation in both cell lines at this time point. In HL-60 cells a similar number of miRNAs were upregulated by both drugs while in T24 no miRNAs were upregulated by 5-Aza-CdR. This immediate downregulatory response specific to the ribo analog might reflect the incorporation of the drug directly into miRNAs as they were being synthesized. On day 8 the pattern changed radically: 5-Aza-CdR deregulated more miRNAs in both cell lines, again indicating the higher potency of this drug to induce heritable changes in expression.

## Discussion

Within the last year Vidaza^TM^ (5-Aza-CR) has been shown to prolong overall survival in MDS patients [10]. Accordingly, the drug is now commonly used in high-risk MDS patients that are not eligible for allogeneic stem cell transplantation. Since its mechanisms of action are similar to the deoxy analog Dacogen^TM^ (5-Aza-CdR) (see above), it was surprising that the two drugs caused a different overall survival in the two recent phase III trials in MDS patients [10], [11]. To investigate whether this discrepancy could also be associated with diverse biological actions of the two drugs, involving possible RNA incorporation of 5-Aza-CR, we have analyzed in detail the effects of azanucleosides on RNA expression, and find that both coding and non-coding RNA species are deregulated to a significant level by either drug (data summarized in Table 1).

**Table 1:** Comparison of 5-Aza-CR (RNA & DNA Incorporation) with 5-Aza-CdR (DNA only).

Cell type	Drug	Immediate (Day 2)	Heritable (Day 8)
HL60 Cells	5-Aza-CR	⇑mRNA, ↑↓miRNAs	⇑mRNA, ↑CTA, ↑↓miRNAs
	5-Aza-CdR	**⇑**mRNA, **⇑**CTA, ↑↓miRNAs	**⇑**mRNA, **↑**CTA, **↑↓**miRNAs
T24 Cells	5-Aza-CR	↑↓mRNA, **⇓**tRNA*, ↑**⇓**miRNAs	↑mRNA, **↑**CTA, ↑↓miRNAs
	5-Aza-CdR	↑mRNA, ↑CTA,	⇑mRNA, **⇑**CTA, ↑↓miRNAs

Up: **⇑**>⇑>**↑**>↑ Down: **⇓**<⇓<**↓**<↓ * Indirect evidence.

The main biological diversities between the two drugs are observed on day 2 after treatment. At this time point, 5-Aza-CdR upregulates a set of genes involved in oncogenic pathways, however, the most striking finding is the immediate effect of 5-Aza-CR on RNA metabolism, which was also recently shown by others [30]. This may lead to early disruption of many pathways both via the direct influence on protein synthesis, and indirectly via a remarkable downregulation of miRNAs. The effect on RNA metabolism seems to increase with higher doses of the drug. Whether these early differences in RNA expression may also relate to the variable success of treatment by the drugs in patients is uncertain, given that the heritable changes may probably most effectively maintain a clinical response.

In contrast to what has previously been described [26], [30], our data show that an overlapping set of mRNAs was upregulated by the two drugs, particularly 8 days after the initiation of treatment. We observed that most genes upregulated by 5-Aza-CR also become derepressed by 5-Aza-CdR at equitoxic doses. 5-Aza-CR upregulates more genes on day 8 as compared to day 2 in both the hematopoietic (HL-60) and the solid cancer (T24) cell line. Gene reactivation seems to be progressive with time which may in part explain the differences between our results and the previous studies [26], [30], in which gene expression was analyzed immediately after 72 hours and 48 hours of continuous treatment of different cell lines.

The two FDA approved hypomethylating drugs are generally thought to work by reactivating the transcription of TSGs that have been downregulated by promoter hypermethylation in cancer. Many *in vitro* studies have shown that both drugs induce transcription of methylated TSGs through hypomethylation of the gene promoters [31]. Quite surprisingly, our array data does not show a significant upregulation of the methylated TSGs in either of the two cell lines. So, although these genes may be upregulated to levels detectable by highly sensitive PCR-based methods, they are not part of the *general dominant* expression pattern induced by these drugs. Mechanisms other than TSG derepression may therefore also influence drug activity. Interestingly, a recent study of primary MDS patients treated with a combination of 5-Aza-CR and an HDAC inhibitor, also demonstrated that on day 15 after treatment, only a few samples showed a significant upregulation of TSGs, even when analyzed by PCR based methods, and TSG upregulation could not be used as a measure of treatment efficacy after 4 cycles [32]. Our data showed that the main common feature of the two drugs was to induce a set of CTAs as has previously been pointed out [33]. Some of these have CpG islands in their gene promoters [34], and for the *XAGE1D* promoter hypomethylation was associated with gene transcription. However, a proportion of the upregulated CTAs did not have promoter CpG islands. CTCFL/BORIS was one methylated CTA that became significantly upregulated in both cell lines by both drugs. CTCFL/BORIS is normally only expressed in germinal cells and embryonic stem cells. When expressed in somatic cells it may antagonize the activity of the epigenetic regulator CTCF, which is ubiquitously expressed in all somatic cells. CTCF is a highly conserved epigenetic controller that, depending on the cellular context, may be involved in gene activation, repression, enhancer blocking, insulation, genomic imprinting and long-range chromosomal interactions [35]. There is now evidence that CTCF is involved in creating large inter- and/or intra chromosomal loops that may protect larger chromosomal segments from transcription [36]. The upregulation of CTCFL/BORIS in both cell lines (Fig. 3 and supplementary Fig. S4) suggests that many of the normal CTCF activities may be interfered with during 5-Aza-CR and 5-Aza-CdR treatment. Accordingly, gene segments that are normally controlled by CTCF like some CTAs and the imprinted H19 and DLK1 genes become upregulated. For the majority of CTAs, upregulation coincided with upregulation of CTCFL/BORIS by both treatment modalities, indicating that CTCFL/BORIS may in part be responsible for the reactivation of CTAs as have previously been described for the MAGE-A1 antigen [24], [37]. Since CTAs are normally only expressed at immune privileged sites like the testis, they are not subjected to self-tolerance. Accordingly, CTAs may stimulate an immune response when expressed in somatic cells, and may elicit coordinated humoral and cell mediated immune responses [38].

A central difference between the two drugs is that only the ribo analog exerts downregulation of RNA on day 2. All RNA species are downregulated, but this appears to be distinctive for small RNA species such as miRNAs and tRNAs. 5-Aza-CR seems to have an immediate downregulatory effect on mRNAs, particularly at higher doses (as used in the T24 cells) and on miRNAs at both lower and higher doses in both cell lines. In T24 the downregulation of miRNAs is dramatic with 46 miRNAs down on day 2, and this is even more pronounced with higher doses (data not shown). Since one miRNA may have several hundred potential mRNA targets, downregulation of miRNAs may be crucial to a large number of cellular functions [39].

These observations may be directly linked to the fact that 5-Aza-CR treated T24 cells show significant upregulation of tRNA synthetases on day 2, a change that is not seen by 5-Aza-CdR, which show a general tendency towards tRNA synthetase downregulation. These data are in line with the recent observations by Hollenbach et al [30]. On day 8 both drugs in both cell lines show tRNA synthetase downregulation. It was recently shown that 5-Aza-CR, but not 5-Aza-CdR, directly inhibits the RNA methyltransferase DNMT2, thereby causing hypomethylation of tRNA, which may probably lead to its destabilization [40]. Accordingly, it is tempting to speculate if the incorporation into RNA and DNMT2 inactivation cause a general, immediate decrease of certain RNA species. Furthermore, it has been shown that the tRNAs produced during 5-Aza-CR treatment are less efficient in protein synthesis [41]. In this perspective upregulation of tRNA synthetases is most likely a compensatory reaction against the immediate break down of components involved in protein synthesis.

In general, our data show that in both cell lines there is a prolonged induction time in 5-Aza-CR treated cells as compared to 5-Aza-CdR treated cells. This may simply relate to the fact that 5-Aza-CR needs to be converted by ribo nucleotide reductase to obtain maximum activity while 5-Aza-CdR does not. However, other explanations of the prolonged induction time may relate to mechanisms unrelated to DNA methylation such as induction of transcription factors including CTCFL that secondarily upregulates CTAs. Alternatively, the immediate downregulation of tRNAs or CTA inhibitory miRNAs may be of importance.

The fact that the drugs have certain distinct immediate activities on the transcriptome may suggest they can be used sequentially or even in combination therapy. This is supported by a recent study showing that 5-Aza-CdR resistant cell lines with mutations in the *DCK* gene that encodes deoxycytidine kinase show 5-Aza-CR sensitivity [42]. One clinical investigation of a small patient sample has shown response to 5-Aza-CdR after failure to 5-Aza-CR [43].

However, the data in the present study are solely based on *in vitro* observations and should be taken with precaution. Only sequential measurements of the drug activities during the treatment of patients will disclose which biological reactions are active *in vivo* and crucial for drug efficacy. Thus, the true answer, as to which drug is the most efficient, will require a carefully designed large clinical trial that directly compares the two drugs and their biological attributes.

## Supporting Information

Figure S1 A representative M-FISH karyogram of the HL-60 cell line
(0.04 MB TIF)

Figure S2 Apoptosis analysis by Annexin V/FITC and Propidium Iodide (PI) and cell cycle analysis with EUD/7-AAD assays
(0.88 MB TIF)

Figure S3 Comparison of TSG upregulation in HL-60 by array and RT-qPCR analysis
(0.84 MB TIF)

Figure S4 RT-qPCR validation of CTA expression
(0.51 MB TIF)

Table S1 Primers used for RT-qPCR validation
(0.03 MB DOC)

Table S2 GSEA comparing untreated and treated HL-60 and T24 cells on day 2 and 8, respectively
(0.04 MB DOC)
