# Supplementary figures and images for: Equitoxic Doses of 5-Azacytidine and 5-Aza-2′Deoxycytidine Induce Diverse Immediate and Overlapping Heritable Changes in the Transcriptome
Source: PLoS One. 2010 Sep 29;5(9):e12994. doi: 10.1371/journal.pone.0012994 (PMC2947512; doi:10.1371/journal.pone.0012994)

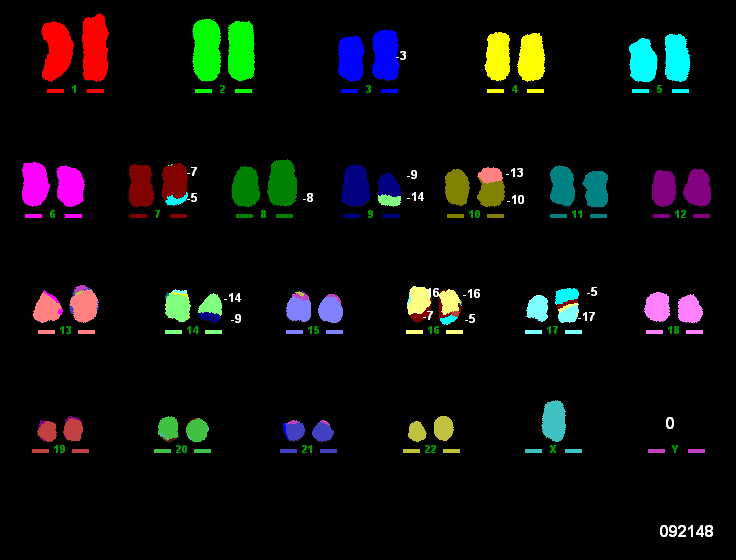

Supplement: Figure S1 — A representative M-FISH karyogram of the HL-60 cell line (0.04 MB TIF) [file pone.0012994.s001.tif]

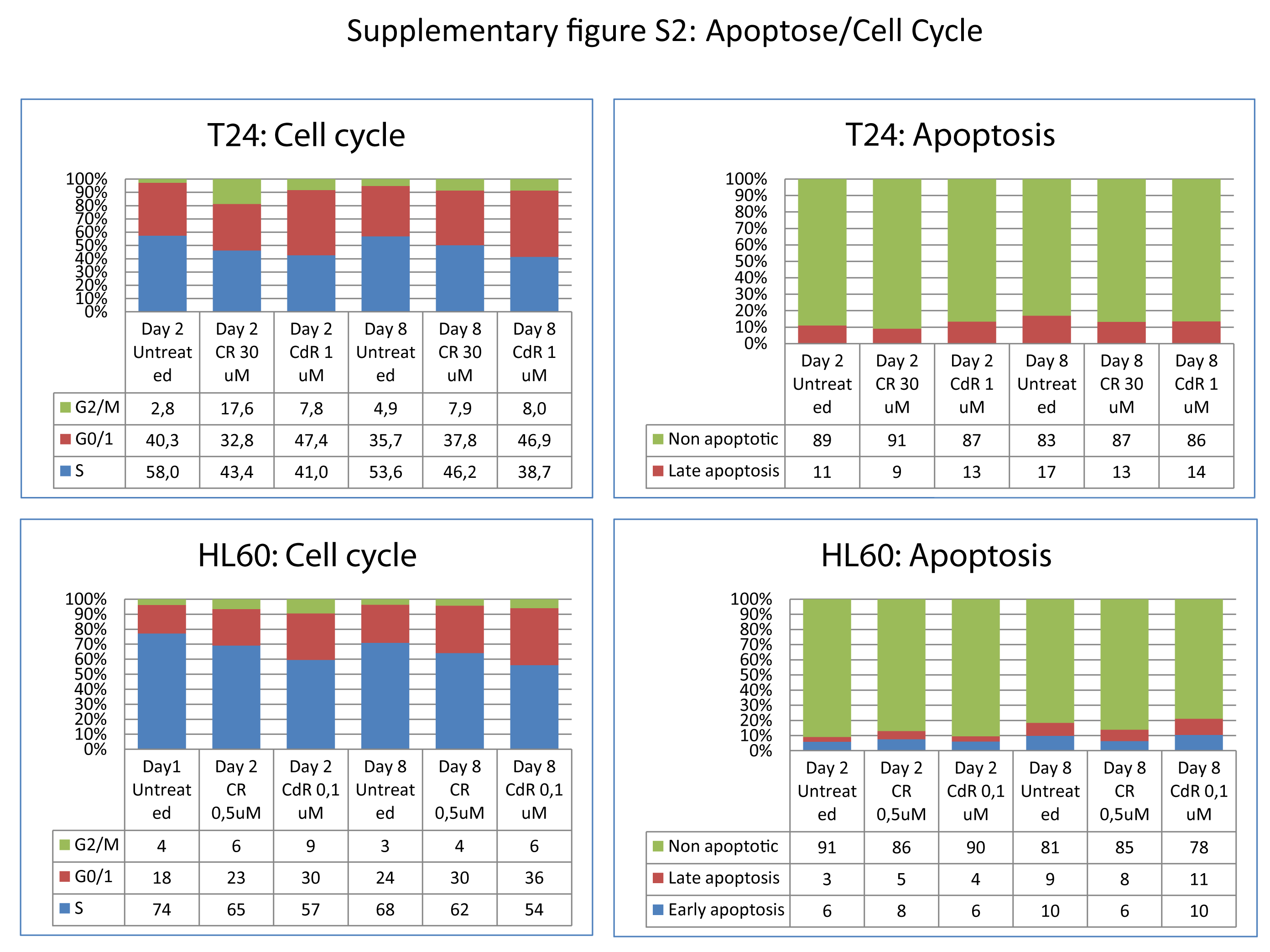

Supplement: Figure S2 — Apoptosis analysis by Annexin V/FITC and Propidium Iodide (PI) and cell cycle analysis with EUD/7-AAD assays (0.88 MB TIF) [file pone.0012994.s002.tif]

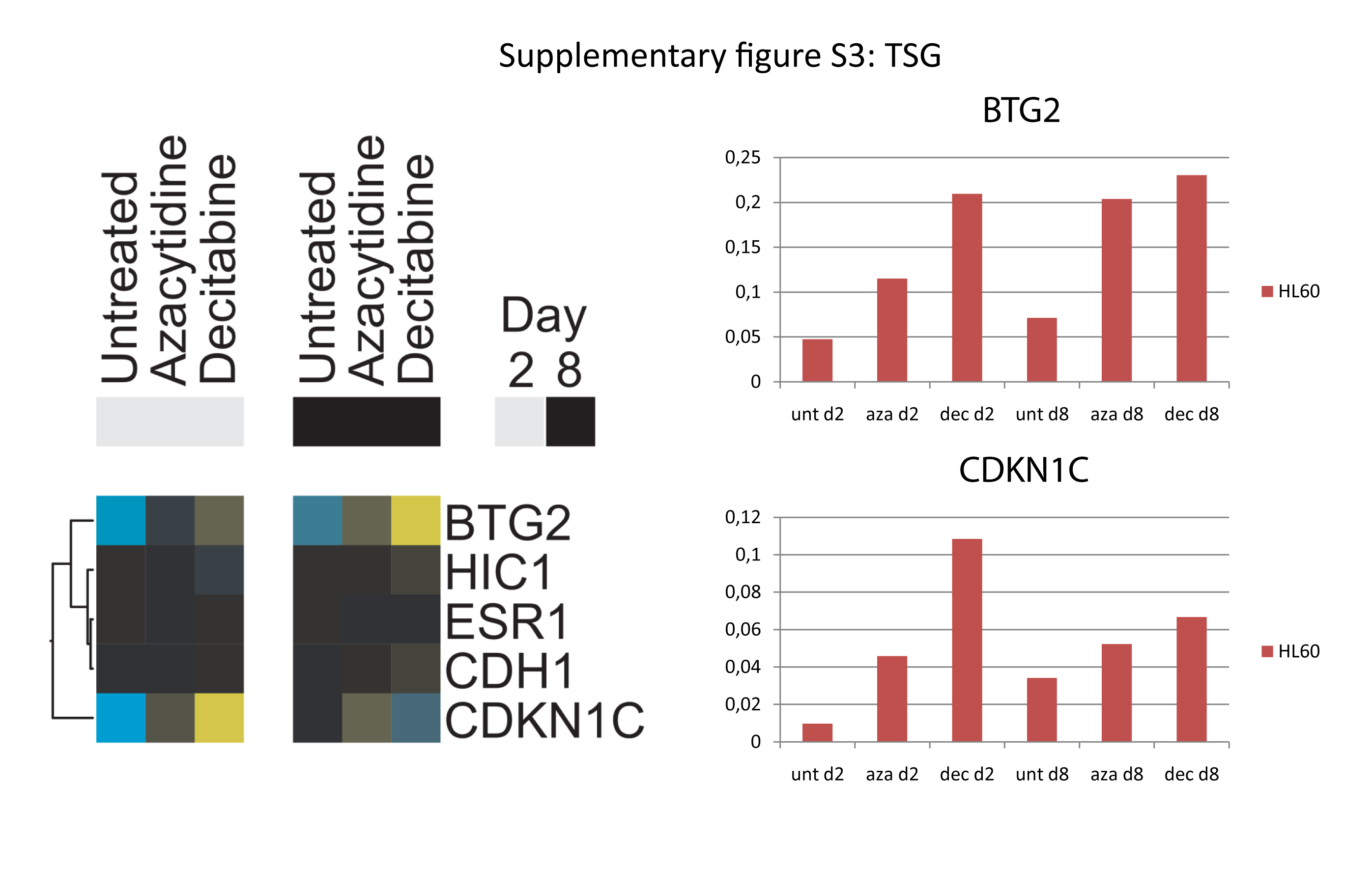

Supplement: Figure S3 — Comparison of TSG upregulation in HL-60 by array and RT-qPCR analysis (0.84 MB TIF) [file pone.0012994.s003.tif]

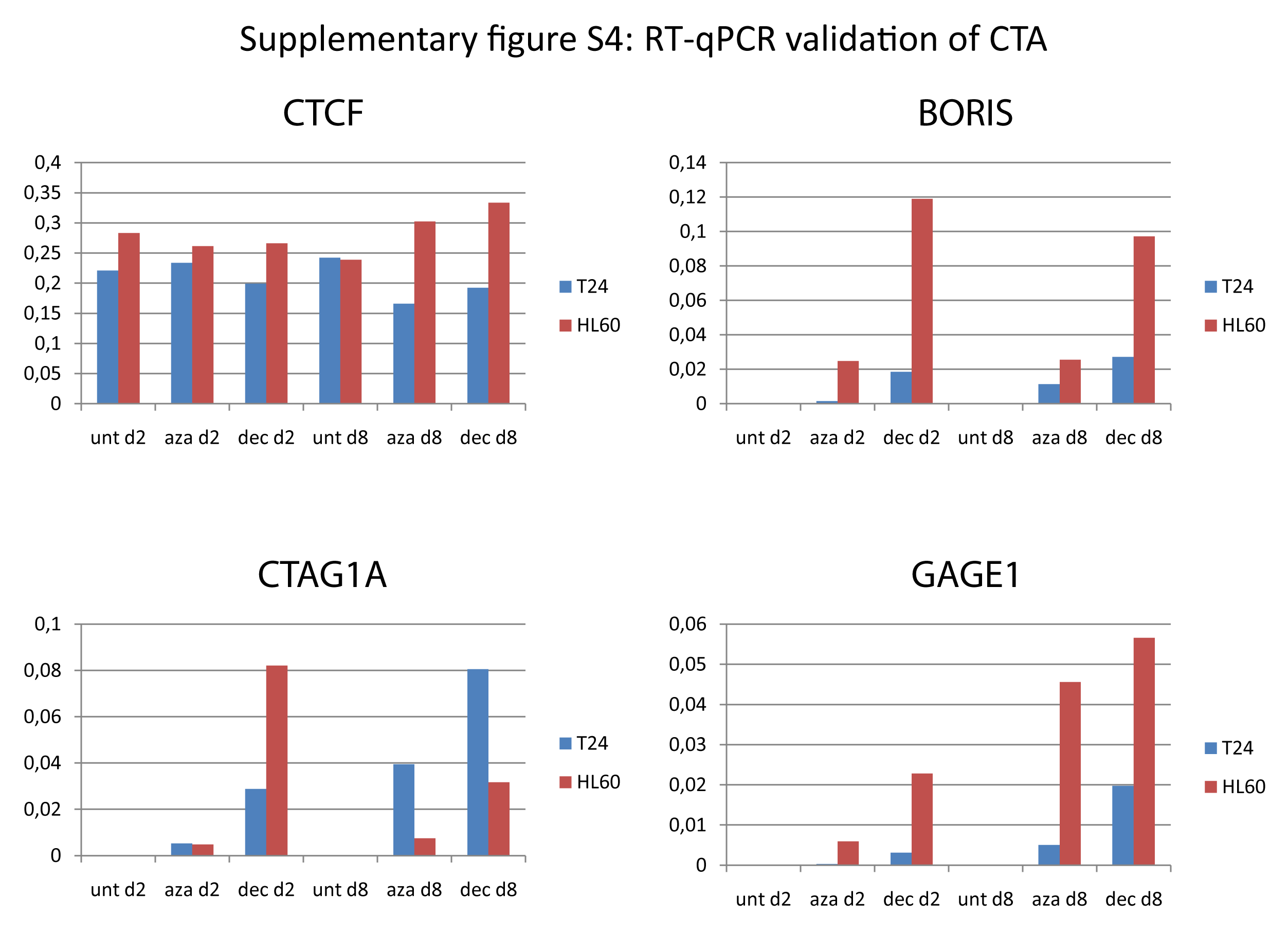

Supplement: Figure S4 — RT-qPCR validation of CTA expression (0.51 MB TIF) [file pone.0012994.s004.tif]
