# Supplementary material for: Equitoxic Doses of 5-Azacytidine and 5-Aza-2′Deoxycytidine Induce Diverse Immediate and Overlapping Heritable Changes in the Transcriptome
Source: PLoS One. 2010 Sep 29;5(9):e12994. doi: 10.1371/journal.pone.0012994 (PMC2947512; doi:10.1371/journal.pone.0012994)
Supplement: Table S1 — Primers used for RT-qPCR validation (0.03 MB DOC) [file pone.0012994.s005.doc]

| name | sequence | length | Tm | position |
| --- | --- | --- | --- | --- |
| CTAG1A-F | TGTGTCCGGCAACATACTGAC | 21 | 62.3 | 381-401 |
| CTAG1A-R | CAGAAAGCACTGCGTGATCCA | 21 | 62.9 | 501-48 |
|  |  |  |  |  |
| CTCF-F | TGCGGAAAGTGAACCCATGAT | 21 | 62.0 | 441-461 |
| CTCF-R | CCCCTTGTTCTAGTGTCTCCA | 21 | 60.5 | 544-524 |
|  |  |  |  |  |
| CTCFL-F | AGTGACGAAATTGTTCTCACAGT | 23 | 60.1 | 634-656 |
| CTCFL-R | GGTTCCTTTTGCTCCCTTTGT | 21 | 60.7 | 768-748 |
|  |  |  |  |  |
| B2M-F | TGCTGTCTCCATGTTTGATGTATCT | 25 | 54.32 | 589 613 |
| B2M-R | TCTCTGCTCCCCACCTCTAAGT | 22 | 55.87 | 674 653 |
|  |  |  |  |  |
| GAGE1-F | ATCGACCTATCGGCCTAGACC | 21 | 62.4 | 18-38 |
| GAGE1-R | GATCCTGACGTTGAGTTGCTG | 21 | 61.0 | 154-134 |
|  |  |  |  |  |
| GAPDH-F | CATGAGAAGTATGACAACAGCCT | 23 | 60.0 | 409-431 |
| GAPDH-R | AGTCCTTCCACGATACCAAAGT | 22 | 60.8 | 521-500 |
|  |  |  |  |  |
| CDKN1C-F | ACATCCACGATGGAGCGTC | 19 | 61.8 | 25-43 |
| CDKN1C-R | GGAAGTCGTAATCCCAGCGG | 20 | 62.4 | 196-177 |
|  |  |  |  |  |
| BTG2-F | CTCCATCTGCGTCTTGTACGA | 21 | 61.6 | 351-371 |
| BTG2-R | AGACTGCCATCACGTAGTTCT | 21 | 60.6 | 469-449 |
