# Supplementary material for: Equitoxic Doses of 5-Azacytidine and 5-Aza-2′Deoxycytidine Induce Diverse Immediate and Overlapping Heritable Changes in the Transcriptome
Source: PLoS One. 2010 Sep 29;5(9):e12994. doi: 10.1371/journal.pone.0012994 (PMC2947512; doi:10.1371/journal.pone.0012994)
Supplement: Table S2 — GSEA comparing untreated and treated HL-60 and T24 cells on day 2 and 8, respectively (0.04 MB DOC) [file pone.0012994.s006.doc]

|  | Day 2 | | | | Day 8 | | | |
| --- | --- | --- | --- | --- | --- | --- | --- | --- |
| Geneset | T24-Aza-CR | T24-Aza-CdR | HL60-Aza-CR | HL60-Aza-CdR | T24-Aza-CR | T24-Aza-CdR | HL60-Aza-CR | HL60-Aza-CdR |
| DNMT1_KO_UP |  |  |  | 0.034 | <0.000 | 0.007 |  |  |
| DNMT1_KO_DN | 0.015 |  |  |  |  |  |  |  |
|  |  |  |  |  |  |  |  |  |
| *Inflammation and immune modulating pathways* |  |  |  |  |  |  |  |  |
| HSA04640_HEMATOPOIETIC_CELL_LINEAGE |  |  | 0.002 | 0.001 |  |  | 0.002 | 0.002 |
| [HSA04060_CYTOKINE_CYTOKINE_RECEPTOR_INTERACTION](http://www.broad.mit.edu/gsea/msigdb/cards/HSA04060_CYTOKINE_CYTOKINE_RECEPTOR_INTERACTION.html) |  |  | 0.007 | 0.044 | 0.021 |  | 0.042 |  |
| TNFA_NFKB_DEP_UP |  |  | 0.023 |  | 0.002 | 0.002 |  |  |
| PASSERINI_INFLAMMATION |  |  | 0.004 |  | 0.009 |  |  | 0.042 |
| BRENTANI_IMMUNE_FUNCTION |  |  | 0.019 | 0.006 | 0.003 | 0.036 | 0.001 | 0.009 |
| DER_IFNB_UP |  |  |  |  | 0.000 | 0.003 |  |  |
| DER_IFNA_UP |  |  |  |  | 0.000 | 0.014 |  |  |
|  |  |  |  |  |  |  |  |  |
| *Diverse pathway^*^* |  |  |  |  |  |  |  |  |
| LEE_MYC_E2F1_UP |  | 0.021 |  | 0.003 |  |  |  |  |
| LEI_MYB_REGULATED_GENES |  | 0.015 |  | 0.007 |  |  |  |  |
| LEE_E2F1_UP |  | 0.023 |  | 0.020 |  |  |  |  |
|  |  |  |  |  |  |  |  |  |
| *Amino acid synthesis* |  |  |  |  |  |  |  |  |
| PENG_LEUCINE_UP | 0.001 |  |  |  |  |  |  |  |
| PENG_GLUTAMINE_UP | 0.039 |  |  |  |  |  |  |  |

* Direct comparison between Aza-CR and Aza-CdR treatment
